# Numbers of professionals at ward reviews and mental health tribunals: an idea by peer workers with lived experience of mental illness for “closed” and “open” meetings

**DOI:** 10.1093/schizbullopen/sgaf025

**Published:** 2025-10-22

**Authors:** Benjamin Gray, Matthew Sisto, Renee Conley, India Sisto

**Affiliations:** Department of Patient Experience, EPUT; Department of Patient Experience, EPUT; Department of Patient Experience, EPUT; Department of Patient Experience, EPUT

## Acute Psychosis: The Need for Closed Meetings

Having a mental health problem can be hard enough; for example, one of the authors has heard threatening voices shouting “Hot fire in your eyes!” and “On the bonfire with him!”. But the ward can also be a distressing and hostile environment with both the mental illness inside the individual (eg, hearing voices) and also the mental illness of other service users (shouting, agitation, and sporadic violence that results in restraint and seclusion). Staff can sometimes be buried in ward routines, such as observation and administration, so can appear detached, disconnected, and even apathetic in their care of people with mental illness. Certainly, psychiatrists and mental health nurses are very time-limited because of their many duties.

Peer work, on the other hand, is based upon connection, empathy, compassion, and reciprocity (eg, giving and taking in conversation). The peer worker’s experience of mental illness connects with the service user’s lived experience of mental illness, which establishes rapport, trust, fuller communication and hence gets a better picture of what is going on in service users’ lives and the stage of their journey in mental illness.

The idea for this article came at first from participant observation by Ben and later from his work with his colleagues and co-authors. Ben noticed the distress of service users before ward reviews and mental health tribunals. On one occasion a service user was shaking with fear and anxiety, having a panic attack before a ward review, so requested tranquilizing medication. On another occasion, a young 18-year-old service user was wringing his hands and crying with distress before his review, much to his mother’s and peer worker’s concern. On another day a service user was becoming agitated, hitting the door of his bedroom and arguing with staff before his mental health tribunal. More recently, Ben noticed that ward reviews are often preceded by fear, agitation, and trembling hands of service users. When this occurs, Ben often calms and reassures people by saying: “You’ll be all right. We’re all on your side.”

Because of the number of professionals and perhaps the disconnection of staff, ward reviews and mental health tribunals become a daunting and hostile environment for people in psychosis. According to three service users:

“Ward reviews frighten me. There are too many people and it’s confusing. I don’t feel they listen to me. It’s a bit like being a rabbit in the headlights.”

“My psychiatrist ignores me and when I try to talk to him he pushes his hand towards me, like he’s pushing me away. I think he’s more interested in my dose of antipsychotics than how I’m feeling. It’s scary and you can’t say everything you want to say. There are too many staff in the room. My peer worker helped calm me down and have my say and said there are advocates and group psychology on the ward, so I felt he really listened to me and really helped.”

“I don’t want to go in there. It’s really crowded and they tell lies about me. I’m not mentally ill. The police couldn’t pin anything on me so they brought me here (the ward).”

On one occasion, Ben counted 12 professionals in a ward review. Ask yourself one question: would you like to talk about your mental distress in front of 12 people you do not know? As the above quotes indicate, this creates a distressing and hostile environment where people and their psychosis are not listened to by psychiatrists, mental health nurses, and other staff.

The authors therefore put forward the idea for “closed meetings” of no more than 3 professionals when the service user is in acute psychosis, agitated, or known to find ward reviews a frightening or hostile environment. Mental health tribunals should also decrease their numbers if this is requested by the person with mental illness.

## Showing Insight: The Need for the Multi-Disciplinary Team and Open Meetings

Conversely, when the person with mental health problems is showing insight or in remission, it is possibly better to have ward reviews or mental health tribunals with the wider multidisciplinary team. As a service user said shortly before he was discharged:

“It’s good to have the full team because they can listen to you and help you more. Give you advice and co-ordinate your care when you leave. My peer worker helped me to think of what I’m going to do next. So, I am going to volunteer at Beacon House (a local homeless charity) and they have Sunday dinners at the Salvation Army. There’s a Quaker House too with support meetings (by MIND)”.

When a person is showing insight into their mental health and in remission, it is perhaps necessary for meetings to be of a larger number of professionals. These help service users in many ways, for example, arranging housing and state benefits; organizing times for tablet medication or depot injections; signposting to services and local groups; and assisting with self-care. At this stage of the service user’s journey in mental health, the focus shifts from someone’s acute or severe psychosis to managing a better life in the community, which requires input and support from the multidisciplinary team.

## Discussion

Peer workers have helped people with mental health problems by advocating for them and encouraging them to advocate for themselves at ward reviews and mental health tribunals. Feedback from staff and service users has been positive, and service users have said it has improved their care and lives while on the ward. Service users can be triggered by ward reviews and tribunals. Peer workers de-escalate service users, advising them of what to say in these contexts and accompanying them to give people feelings of safety.

Peer work involves connection, empathy, compassion, and reciprocity. It is based upon the shared experience of mental illness of both peer workers and service users. Peer workers listen to people’s stories and get to know and understand them at a deeper level than other staff, so they are better able to facilitate their recovery journey and eventual discharge. Peer workers also do recovery plans and next steps for service users (eg, by listening to service users and helping them in what they might ask for at ward reviews and mental health tribunals, especially increased leave).

Through connecting with people and exploring their stories, peer workers are able to give a fuller picture to psychiatrists and mental health nurses of what is really going on in people’s lives and the stage of their journey in the mental health system. Peer work helps service users to feel more comfortable about telling and disclosing difficult and sensitive experiences to psychiatrists and other health staff. Psychiatrists are very time-limited because of their many duties, as are mental health nurses. Healthcare assistants are focused on observation, safety, a stable ward environment, process, and administration. Peer workers make up for this perceived disconnection and provide more holistic and therapeutic care, including one-to-one sessions, hearing voices groups, art and music therapy, managing stress and anxiety or coping with fear groups, and aromatherapy, to mention just a few. They compensate for power imbalances and are service-user (not service) focused.

As a service user said:

“I just want to thank you for all your help and support while I’ve been here (the ward). You can tell that peer workers really care and have got your back. I can’t thank you enough. You have given me a voice”.

Certainly, more research on this topic and open or closed meetings is much needed. It could perhaps be a qualitative approach to capture the depth of people’s experiences in their journey of recovery on mental health wards. It should finally be noted that this article mainly expresses the views of the first author (Ben).

